# Gold Rod-Polyethylene Glycol-Carbon Dot Nanohybrids as Phototheranostic Probes

**DOI:** 10.3390/nano8090706

**Published:** 2018-09-10

**Authors:** Yuefang Niu, Guo Ling, Li Wang, Shanyue Guan, Zheng Xie, Eran A. Barnoy, Shuyun Zhou, Dror Fixler

**Affiliations:** 1 Key Laboratory of Photochemical Conversion and Optoelectronic Materials, Technical Institute of Physics and Chemistry, Chinese Academy of Sciences, 29 Zhongguancun East Road, Haidian District, Beijing 100190, China; niuyuefang15@mails.ucas.ac.cn (Y.N.); lingguo14@mail.ipc.ac.cn (G.L.); wangli165@mails.ucas.ac.cn (L.W.); guanshanyue@mail.ipc.ac.cn (S.G.); 2 Faculty of Engineering and Institute of Nanotechnology and Advanced Materials, Bar-Ilan University, Ramat-Gan 52900, Israel; eabnoy@gmail.com; 3 University of Chinese Academy of Sciences, 19A Yuquan Road, Shijingshan District, Beijing 100049, China

**Keywords:** gold nanorod, carbon dots, nanohybrids, bioimaging, photothermal therapy

## Abstract

Emphasis using phototheranostics has been placed on the construction of multifunctional nanoplatforms for simultaneous tumor diagnosis and therapy. Herein, we put forth a novel nanosized luminescent material using the incorporation of red emissive carbon dots on gold nanorods through polyethylene glycol as a covalent linkage for dual-modal imaging and photothermal therapy. The novel nanohybrids, not only retain the optical properties of the gold nanorod and carbon dots, but also possess superior imaging performance in both confocal laser scanning microscopy and fluorescence lifetime imaging microscopy. The nanohybrids also exhibit excellent photothermal performance as phototheranostic nanohybrid probes for in vitro assays. This study promises a new multifunctional nanoplatform for cancer diagnostics and therapeutics.

## 1. Introduction

Cancer theranostics, which combine bioimaging diagnostics and cancer therapy, have the potential to help millions of people in typically fatal situations [1]. Over the last decade, this field has witnessed the rapid development of nanotheranostic devices. These devices are constructed using both organic and inorganic nanomaterials to integrate both therapeutics and bioimaging agents into one entity, simultaneously realizing their functionalities [2,3].

In theranostic systems, a pivotal concern is the choice of imaging techniques to accurately disclose the location of tumors for a specific diagnosis. Several methods, such as computed tomography (CT), fluorescence (FL) imaging, magnetic resonance imaging (MRI), positron emission tomography (PET), photoacoustic (PA) imaging and upconversion luminescence (UCL) imaging have been applied [4,5,6]. However, each technique has its own inherent limitations. To address their shortcomings, tremendous efforts have been made in the development of multimodal imaging, which can take advantage of different techniques to properly meet clinic requirements [7,8,9].

For instance, despite the excellent sensitivity of FL imaging, its limited tissue penetration depth [10,11] and the finite information derived from fluorescence intensity micrographs of cells have compromised its diagnostic abilities. Meanwhile, fluorescence lifetime imaging microscopy (FLIM) has been proven as a highly advanced spectroscopic method for biological and biomedical applications [12]. The excellent performance of FLIM can contribute to high contrast images that are independent of excitation intensity and fluorophore concentration [13]. Moreover, it provides both temporal and spatial information of intracellular structures labeled fluorescently by detecting changes in the fluorescence lifetime (FLT) [14]. Thus, the integration of FLIM with FL imaging could be more favorable for accurate cancer diagnosis. Therefore, there is a need to find more appropriate fluorescent probes for FLIM and FL imaging.

Fluorescent probes, such as organic fluorescent dyes and quantum dots (QDs), have been widely explored in biomedical fields for imaging [15,16,17,18]. However, the biocompatibility, photo-bleaching and photo scintillation of some probes hinder these applications [19,20]. In contrast, carbon dots (CDs) are ideal candidates for biological applications due to their biocompatibility, chemical inertness, as well as strong fluorescence performance, photochemical stability, and easy functionalization [21,22,23,24,25,26]. In the current era, a range of research aims to investigate the potential of CDs as biocompatible nanoprobes for targeting cancer cells in vitro. For example, CDs doped with heteroatoms (such as N, P, and S) were widely studied for fluorescence imaging in cells [27,28,29]. However, CDs are rarely regarded as a therapeutic agent [30]. Moreover, a variety of photothermal agents (e.g., layered double hydroxides, gold nanorods, chlorin e6) were combined with CDs and the prepared hybrid system was successfully used as a theranostic agent [31,32,33]. Therefore, photothermal agents can be integrated with CDs to achieve multifunctional cancer theranostics.

In photothermal therapy (PTT), near-infrared (NIR) light is applied for the generation of localized heat energy from specifically-designed nanomaterials, which can cause hyperthermia and hence the apoptosis or necrosis of cancer cells [30,34,35,36]. The unique surface plasmon resonance (SPR) of noble metal nanoparticles, especially gold nanoparticles, promote their ability to quickly and effectively convert absorbed photon energy into heat in the picosecond time domain [37]. In this catalog, gold nanorods (GNRs) have been extensively studied due to their facile synthesis and surface modification, biocompatibility, superior tunable optical properties and photostability, good cellular affinity and long blood circulation [38]. One unique advantage of GNRs is their longitudinal SPR peak can be adjusted to the NIR region by modulating the aspect ratio (length/width). NIR is known to have optimal light penetration in biological tissues due to its minimal absorption by chromophores and water [39]. Furthermore, the high scattering cross sections of GNRs render them good contrast agents for dark field microscopy imaging.

Early diagnosis and definitive therapy can be integrated into an unprecedented nanoplatform to break the limitations of individual functionality. To date, GNRs were integrated with fluorescent dyes [14] or quantum dots (QDs) [40], either by electrostatic interaction [32] or covalent linkages [41], for their utility as an imaging contrast agent. However, there is still a need for new agents with a stable structure, excellent biocompatibility, and high therapeutic efficiency to meet the demands for clinical applications. Polyethylene glycol (PEG) is a biocompatible polymer that has been used for an extremely wide range of products, ranging from skin care products to tablet formulations, laxatives, and food additives [42]. Thus, PEG is the most suitable material for latent clinical applications. In addition, these biocompatible PEG chains can be easily functionalized as a covalent linker, which can improve the chemical stability of the hybrids in physiological environments, as well as prevent absolute quenching of CD fluorescence when bound to GNRs.

In this paper, we report the construction of a covalently-linked nanohybrid as a novel nanoplatform for dual-modal imaging and phototherapy made up of GNRs and CDs bridged with PEG (GNR–PEG–CDs), and these luminescent nanohybrid materials express excellent fluorescence features for FLIM and confocal FL imaging, leading to good spatial resolutions and a strong response to PTT. In addition, they have a low toxicity and good biocompatibility. This study exhibits a new multifunctional nanoplatform, i.e., GNR–PEG–CDs, for cancer diagnostics and therapeutics.

## 2. Materials and Methods

### 2.1. Materials

HAuCl_4_·3H_2_O and sodium borohydride (NaBH_4_), were purchased from J&K Chemical. Co. (Beijing, China). Cetyltrimethyl Ammonium Bromide (CTAB), L-ascorbic acid, HCl, silver nitrate (AgNO_3_) and *p*-phenylenediamine were purchased from Sinopharm Chemical Reagent Co., Ltd. (Beijing, China). Thiolated and methoxyl terminated polyethylene glycol (mPEG-SH, molecular weight 1000 Da), Thiolated and carboxyl terminated polyethylene glycol (SH-PEG-COOH, molecular weight 1000 Da), were purchased from Peng Shuo Biological Technology Co., Ltd. (Shanghai, China). 1-ethyl-3-(3-dimethyl-aminopropyl) carbodiimide hydrochloride (EDC) and N-hydroxysuccinimide (NHS), were purchased from Aladdin (Shanghai, China). All reagents were of analytical grade and used without further purification. High glucose Dulbecco’s modified Eagle’s medium (DMEM) was purchased from Gibco (Invitrogen, Carlsbad, CA, USA). Cell Counting Kit-8 (CCK-8) was obtained by Dojindo China Co., Ltd. (Shanghai, China). HeLa cells were obtained from the Institute of Basic Medical Sciences Chinese Academy of Medical Sciences (Beijing, China).

### 2.2. Preparation of Gold Nanorods

The seed-mediated growth method was utilized to prepare GNRs according to Nikoobakht’s method, with some modifications [43]. In this seed solution, ice-cold aqueous NaBH_4_ solution (0.01 M, 0.6 mL) was added to an aqueous mixture solution, which was composed of HAuCl_4_ (0.01 M, 0.25 mL) and CTAB (0.1 M, 9.75 mL), followed by rapid mixing for 2 min. Then, the seed solution was kept at 27 °C for 2 h for use in growth solution. The growth solution was composed of CTAB (0.1 M, 200 mL), HAuCl_4_ (0.01 M, 10 mL), AgNO_3_ (0.01 M, 2.6 mL), HCl (1 M, 5 mL) and L-ascorbic acid (0.1 M, 2.4 mL). Next, 240 µL seed solution was injected into the growth solution, and was allowed to incubate at 30 °C for a period of 24 h. The resultant GNRs solution was then centrifuged and redispersed in water several times to remove the unbound excess surfactant. Finally, the GNRs were obtained and resuspended in water for further use.

### 2.3. Preparation of GNR–PEG

The protocol was adapted and modified from previous reports [14]. In short, 90% thiolated mPEG (mPEG–SH) and 10% thiolated PEG–COOH (SH–PEG–COOH) were added to a solution of GNR and allowed to incubate for 2 h at room temperature before removing excess thiolated PEG by centrifugation to afford GNR-PEG. Three different molecular weights of PEG (400, 600, 1000) were implemented in this process.

### 2.4. Preparation of GNR–PEG–CDs Nanohybrids

The CDs were prepared using a modified protocol by Jiang et al. [44] in which 0.5 g *p*-phenylenediamine was dissolved in 20 mL of water at 180 °C for 12 h in a polytetrafluoroethylene-lined stainless autoclave and then purified using silica gel column chromatography after cooling to room temperature. The resultant CDs were dispersed in water and stored at 4 °C for the further use. Subsequently, 100 μL of 10 mM EDC and NHS were added to 10 mL of the GNR–PEG solution and mixed for 15 mins; then, 1 mL of CD solution was added. The solution was left to stir for 12 h at room temperature. In addition, the aqueous solution was dialyzed against 2000 mL of water for 48 h with 6 changes.

### 2.5. Measurement of Photothermal Performance

A MDL-III-808 nm model laser was used in the photothermal experiment, in which the light source was a NIR laser of 808 nm. The specific experimental steps and parameters were as follows: 1.5 mL of each of phosphate-buffered saline (PBS), an aqueous solution of CDs, GNR–PEG and GNR–PEG–CDs were introduced in a quartz cuvette and this was followed by irradiation treatments with the 808 nm laser, in sequence. The laser power output at the light source was 1.5 W cm^−2^. The temperature of the liquid in the quartz cuvette was recorded with a digital thermometer equipped with a thermocouple probe under irradiation. In addition, to prove the thermal and mechanical stability of the hybrid, we provide the electron microscopic images of the hybrid after irradiation and ultrasound for 10 min. An ultrasonic machine (KQ5200DE) (Kunshan Ultrasonic Instruments Co., Ltd., Kunshan, China) was used to perform the ultrasound.

### 2.6. Cytotoxicity

HeLa cells were cultured in Dulbecco’s modified Eagle medium (DMEM) with 1% penicillin-streptomycin and 10% fetal bovine serum (FBS). The cells (1 × 10^4^ cells per well) were seeded into a 96-well plate for 24 h at a humidified atmosphere maintaining 5% CO_2_ at 37 °C. Then, various concentrations of sample the solutions were added to replace the culture media and subsequently incubated at 37 °C. After further incubation for 24 h, the medium was replaced with 10% CCK-8 reagent solution and incubated for 2 h at 37 °C. The cell viability was calculated as the ratio of the absorbance of the wells. The absorbance at 450 nm was measured by a microplate reader (Multiskan FC, Thermo Scientific, Waltham, MA, USA).

### 2.7. Confocal Fluorescence Imaging Measurements

Confocal fluorescence imaging measurements were performed on HeLa cells with Hoechst staining. Hoechst is a nuclear dye of which optimal excitation wavelength is 350 nm, and the emitted wavelength is 460 nm. Briefly, HeLa cells (1 × 10^5^ cells per well) were seeded onto a plate for 24 h at 37 °C, followed by incubation with pristine CDs and GNR–PEG–CDs for another 24 h. Subsequently, the cells were washed with PBS three times and stained with Hoechst. Subsequently, CDs and GNR–PEG–CDs were cultured with HeLa cells to perform confocal imaging measurements. A dual-channel imaging mode was used in this test. Channel I used a 405-nm wavelength laser as the excitation light to excite Hoechst, and the location information of the nuclei stained by Hoechst was collected. Channel II was stimulated by a laser of 485 nm wavelength to get information of CDs and GNR–PEG–CDs.

### 2.8. FLIM Measurements

The GNR–PEG–CDs sample was measured in terms of HeLa cells to observe the changes in fluorescence lifetime and imaging effect. In this test, the dual-channel imaging mode was used, that is, Channel I used the 405-nm wavelength laser as the excitation light, and an emission light of 482 nm (±35 nm) was collected; Channel II was stimulated by a laser with a 485-nm wavelength and an emission light of 550 nm (±49 nm) was collected.

### 2.9. Sample Characterization

Transmission electron microscopy (TEM) images and high-resolution (HR) TEM were obtained using a HT7700 biological transmission electron microscope (Hitachi, Tokyo, Japan) with an accelerating voltage of 100 kV, and a JEM-2100F high-resolution transmission electron microscope (JEOL, Tokyo, Japan) with an accelerating voltage of 200 kV. Fourier transform infrared (FT-IR) spectra were obtained by an Excalibur HE 3100 (Varian, Palo Alto, CA, USA) in the range from 4000 to 400 cm^−1^. The ultraviolet (UV–vis) absorption spectra were obtained from a U-3000 (Hitachi, Tokyo, Japan). A Cary Eclipse spectrophotometer (Varian, Palo Alto, CA, USA) was used to record the fluorescence spectra and relative quantum yield (QY) values. Rhodamine B (RhB) was used as reference to measure the QY values of CDs and the hybrids. More details about the method can be found in Reference [44] and the Supplementary Materials (SM). Confocal fluorescence imaging and fluorescence lifetime imaging were performed using confocal laser scanning microscopy (CLSM, Nikon, Tokyo, Japan) and a Nikon two-photon fluorescence lifetime imaging microscope (ARsiMP-LSM-Kit-Legend Elite-USX, Picoquant, Berlin, Germany).

## 3. Results and Discussion

### 3.1. Synthesis of GNR–PEG-CDs

**Scheme 1:**
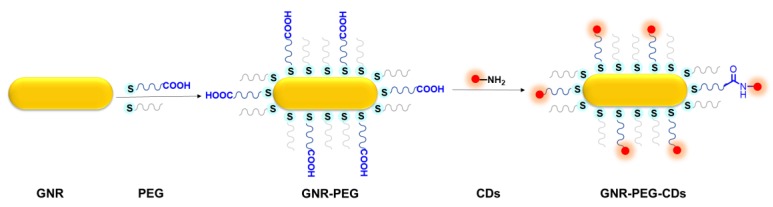
Schematic illustration of GNR–PEG and GNR–PEG–CDs.

**Figure 1:**
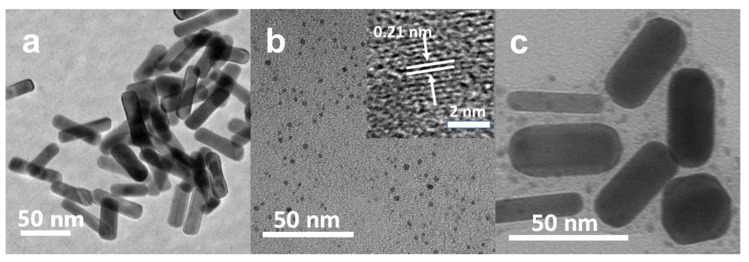
TEM images of GNRs (**a**), CDs (**b**) and GNR-PEG-CD (**c**), inset of (b) is high-resolution (HR) TEM image of CDs.

Scheme 1 demonstrates the preparation protocol of GNR–PEG–CDs. Figure 1 shows TEM images of GNRs, CDs and GNR–PEG–CDs. GNRs, with an average aspect ratio (length/diameter) of ~3.5, were synthesized (Figure 1a) via the seed-growth method. To prevent an absolute quenching effect of fluorescence of the CDs, aggregation of GNRs [45], and, most importantly, to achieve covalent linkage between GNRs and CDs; PEG-SH was grafted onto the surface of GNRs to form highly-monodispersed GNR-PEG. To verify the synthetic results and choose a proper length of chain for PEG, three different molecular weights of PEG (400, 600, and 1000) were tested and evaluated using Zeta potential and UV-vis spectra. The results of the Zeta potential are shown in Figure S1. The potential of GNRs was 45.8 mV. In contrast, the potentials of GNR–PEG_400_, GNR–PEG_600_ and GNR–PEG_1000_ were 44.0, 19.5 and 14.3 mV, respectively. We found that GNR–PEG had a lower Zeta potential than GNR, especially GNR–PEG_600_ and GNR–PEG_1000_. The differences in Zeta potentials are attributed to cationic CTAB being replaced by PEG on the GNR surface. Subsequently, absorption peaks in the UV-vis spectrum were measured. As shown in Figure S2, the SPR peak of the GNRs is centered at 776 nm, which is in the NIR region. While the peaks at 789, 798 and 804 nm were attributed to GNR–PEG_400_, GNR–PEG_600_ and GNR–PEG_1000_, respectively, revealing that the SPR of GNR red-shifts after PEG was grafted onto GNRs. Movements in the position of maximum absorption became much more obvious with an increase in PEG molecular weight, which can be attributed to an increase in the local refractive index of the medium surrounding the GNRs [46]. The above results indicate that PEG was successfully grafted on the surface of GNRs. Hence, GNR–PEG_1000_ was detailed in the following study.

**Figure 2:**
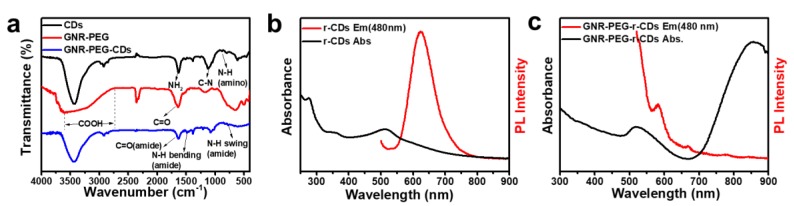
(**a**) The Fourier transform infrared (FT-IR) spectra of CDs, GNR-PEG and GNR-PEG-CDs; (**b**) the UV-vis and PL spectra (excited at 480 nm) of CDs; (**c**) the UV-vis and PL spectra (excited at 480 nm) of GNR–PEG–CDs.

CDs were prepared through a hydrothermal method with *p*-phenylenediamine as a carbon source, and separated by silica gel column chromatography. The TEM images presented in Figure 1b reveal that the sample was well dispersed in water, with an average particle size of approximately 2.1 nm (Figure S3). The HRTEM images further show that CDs exhibit identical lattice fringes with a spacing of 0.21 nm, corresponding to (100) in-plane of graphene. Figure 2b reveals that CDs have an intense UV-vis absorption peak at 514 nm and exhibit a strong red fluorescence emission at 625 nm when excited at 480 nm. By taking RhB as a reference, photoluminescence (PL) QYs of the CDs were estimated to be 16%.

Finally, the GNR–PEG–CDs conjugate was prepared by coupling PEG and CDs with amide bond (Scheme 1 and Figure 1). During the synthesis of GNR–PEG, SH–PEG–COOH was mixed with mPEG-SH in a 1:9 molar ratio and thereby CDs were able to be conjugated on a polymer layer of GNR–PEGs. As depicted in Figure 1c, CDs are surrounding the GNRs at a distance between 6 to 20 nm according to the molecular weight of the spacer. To further verify the hybridization of GNR-PEG-CDs, FT-IR spectroscopy was performed, and the results are presented in Figure 2a. FT-IR showed that the O–H vibration was from 2739 cm^−1^ to 3600 cm^−1^ and the C=O stretching vibration of intramolecular hydrogen bonding was at 1633 cm^−1^ from the carboxyl group of GNR–PEG. This shows that the bending vibrations of the N–H bond in the primary amine group of CDs are at 881cm^−1^ and 1626 cm^−1^. Furthermore, the peak at 1118 cm^−1^ belongs to the C–N stretching vibration from the fatty amine of CDs. Noticeably, it revealed that the stretching vibration of the C–H bond is at 1633 cm^−1^, the bending vibration of the N–H bond is at 1550 cm^−1^ and the swing vibration of the N–H bond is at 772 cm^−1^ for GNR–PEG–CDs. The results infer the interaction, i.e., secondary amide bonding between the amine functionalized CDs and carboxylic acid terminated PEG. These data confirm that functionalization of CDs onto the surface of GNR was successful. One key point in designing new multifunctional comparative materials is to maintain the unique properties of each component. Therefore, the optical properties were investigated. UV-vis absorption spectra (Figure S4) showed that the nanohybrids retained the plasmon resonance absorption feature of GNRs, showing two absorption peaks at 859 and 519 nm. Notably, the long absorption wavelength had a large bathochromic shift of about 83 nm when compared to GNRs. Additionally, GNR-PEG-CDs exhibited a luminescent emission peak at 580 nm when being excited at 480 nm, as shown in Figure 2c. By taking RhB as a reference, PL QYs of the GNR–PEG–CDs were only 1%. The reason for the decrease of PL QYs may be the quenching effect, considering the following points: First, CDs as an energy donor can transfer energy to an acceptor (GNR), constructing a luminescence resonance energy transfer (LRET) system [47]. Second, the stronger optical absorption of GNR than that of CDs can enhance the quenching effect of integrated hybrid materials. In addition, a larger surface area and higher surface energy of GNR can provide more quenching site and thus improve the quenching efficiency [45]. Meanwhile, the distance is close enough to cause emission quenching from LRET. This effect can be explained by changes in the phase of the reflected field with distance and the effects of the reflected near-field on the fluorophore [48].

### 3.2. Temperature Evaluation in Solutions

**Figure 3:**
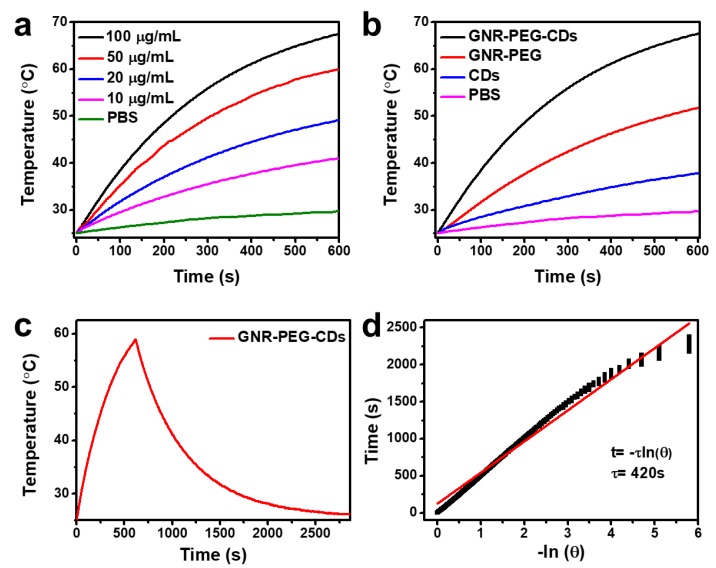
(**a**) The concentration-dependent temperature elevation; (**b**) a time-dependent increase in the temperature; (**c**) photothermal effect of GNR-PEG-CDs; (**d**) the time constant for heat transfer from the solution is determined by applying the linear time data from the cooling stage in (c) versus the negative natural logarithm of the driving force temperature. Samples were carried out under 808 nm (1.5 W cm^−2^) irradiation.

The photothermal properties of GNR–PEG–CDs were evaluated by measuring the temperature change of GNR–PEG–CDs aqueous dispersion under an irradiation of a 808-nm laser at a power of 1.5 W cm^−2^. Then, 1.5 mL of GNR-PEG-CDs dispersion with various concentrations from 0 to 100 μg mL^−1^ of the whole nanoprobe were injected into the quartz cuvette. After consistent exposure for 10 min, the real-time temperature was recorded with a digital thermometer equipped with a thermocouple probe at a detection sensitivity of 0.1 °C. As shown in Figure 3a, while NIR irradiation only led to a slight change of temperature in PBS buffer solution, i.e., 25.0 to 29.7 °C, the photothermal effect was observed in GNR–PEG–CD dispersion from the significant increase of temperature. The values ranged from 41.0, 49.1, 59.9 and 67.5 °C in accordance with GNR–PEG–CD concentrations of 10, 20, 50 and 100 µg mL^−1^, respectively. It appeared that both a continual irradiation and sufficient concentration were efficient for killing cells. A sufficient concentration is optimal for the best therapeutic effects. Several control experiments were done to clarify prior the performance of GNR–PEG–CDs. For example, the time-dependent increase in the temperature of GNR–PEG–CDs (100 µg mL^−1^), GNR-PEG (95 µg mL^−1^), CDs (5 µg mL^−1^) and PBS are shown in Figure 3b. The results show that SPR in the NIR region of GNR–PEG–CDs led to an incremental increase in temperature to a maximum when compared to either GNR-PEG or CDs alone, indicating the GNR-PEG and CDs can synergistically contribute to the light heat conversion efficiency of the GNR–PEG–CDs solution. Figure 3c presents the photothermal effect of GNR–PEG–CDs illuminated with an 808-nm laser (1.5 W cm ^−2^) for 10 min after shutting off the laser. In addition, the photothermal conversion efficiency of the GNR-PEG-CDs solution was calculated according to Roper’s method, through fitting the cool-down curve of the dispersion (Figure 3d), and was determined to be 33.5% (the details of the calculations are shown in the Supplemental Materials). As such, a noticeably higher efficiency was derived from the nanohybrids of GNR with CDs. Furthermore, the plasmonic absorption peak of the structure is located in the NIR region, and thus, facilitates the heat conversion. The above results indicate that the combination of the gold nanorods and carbon dots evidently amplifies the photothermal efficiency. In addition, TEM images of the hybrid (100 µg mL^−1^) after irradiation and ultrasound for 10 min are shown in Figure S5. The results demonstrate that the structure of the hybrid was stable under the laser irradiation and after the ultrasound, indicating the thermal and mechanical stability of the hybrid.

### 3.3. In Vitro Dual-Modal Imaging and PTT

#### 3.3.1. Confocal Laser Scanning Imaging

**Figure 4:**
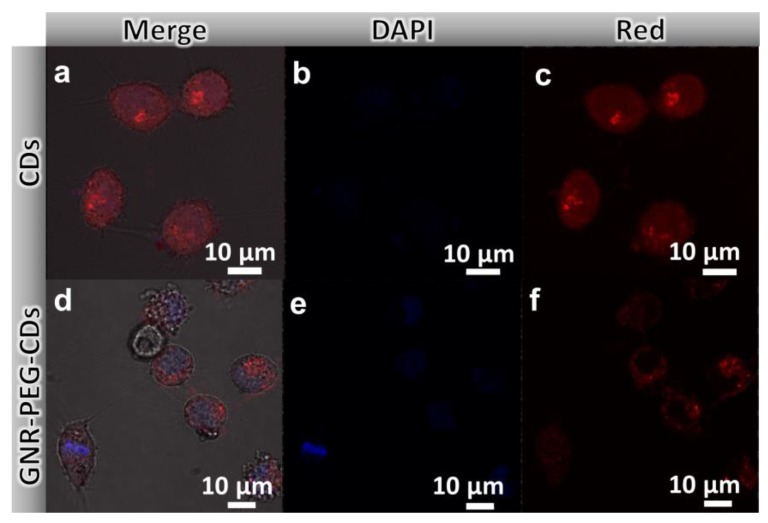
Confocal laser scanning microscopy (CLSM) images of HeLa cells incubated with CDs (**a**,**b**,**c**) and GNR–PEG–CDs (**d**,**e**,**f**) for 24 h under laser excitation of 405 nm (**b**,**e**) and 485 nm (**c**,**f**) respectively. Scale bar, 10 μm.

The remarkable optical properties of GNR–PEG–CDs make it a suitable agent for cancer imaging. First of all, we examined the incubation of the probes with HeLa cells. CLSM images exhibit that the GNR–PEG–CDs nanorods could be efficiently taken up into the cells and distributed mainly in the cytoplasm region (Figure 4d–f). Comparably, free CDs are distributed in both the cytoplasm and the nucleus regions (Figure 4a–c).

#### 3.3.2. Fluorescence Lifetime Imaging

**Figure 5:**
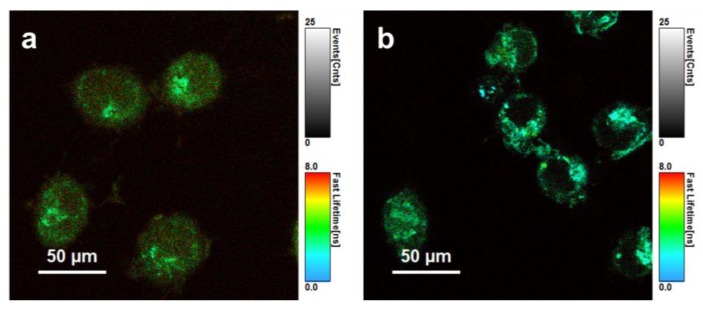
FLIM images of HeLa cells incubated with CDs (**a**) and GNR-PEG-CDs (**b**) for 24 h. Scale bar, 50 μm.

The FLIM measurement was performed in the HeLa cells, a double-component lifetime model was required (Figure 5a,b). Moreover, in the case of the CDs (both with and without GNRs), the FLIM images displayed a significant change in FLT. Visually, the two FLTs of CDs were τ_1_ = 1.8 ns and τ_2_ = 5.9 ns, while those of GNR–PEG–CDs were τ_1_ = 1.6 ns and τ_2_ = 4.3 ns. We observed a substantial decrease in the FLT of GNR–PEG–CDs in cells, which is due to the proximity between the GNRs and the CDs. These results reflect both the quenching effects of GNRs on the FLT of the CDs, as well as the microenvironments of the HeLa cells. In addition, FLIM imaging of the CDs allows for the localization of hybrids within cancer cells.

#### 3.3.3. In Vitro Biocompatibility and PTT

**Figure 6:**
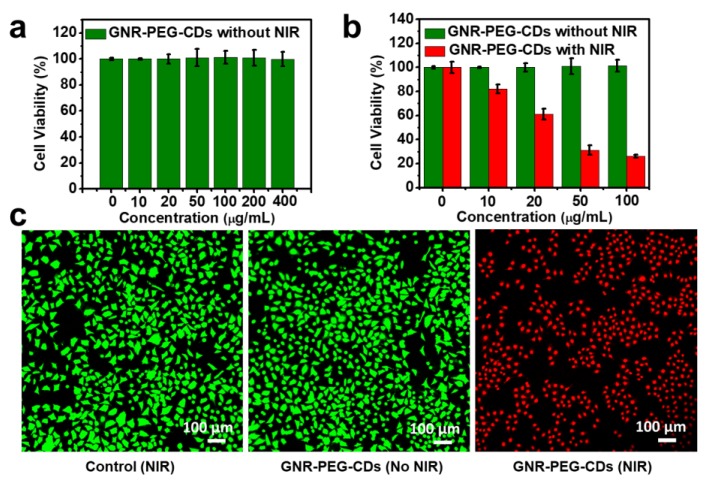
(**a**) In vitro viability results of HeLa cells treated using GNR-PEG-CDs without lasers irradiation (from 0 to 400 µg mL^−1^); (**b**) in vitro viability results of HeLa cells treated with GNR-PEG-CDs (from 0 to 100 µg mL^−1^); (**c**) CLSM images of GNR-PEG-CDs (100 µg mL^−1^) before and after NIR irradiation with Calcein-acetoxymethylester and propidium iodide (PI) co-staining. Scale bars: 100 μm.

The biocompatibility and PTT potential of GNR–PEG–CDs were investigated in HeLa cells. As shown in Figure 6a, without laser irradiation, cell viability remained at almost 100% with GNR–PEG–CDs at a concentration of 400 µg mL^−1^, which represents good biocompatibility of the nanomaterial. It was found that irradiation of HeLa cells at 808 nm caused serious cell death in the case of GNR–PEG–CDs (Figure 6b). The cell viability decreased by about 20% with GNR–PEG–CDs concentration of 10 µg mL^−1^. When the concentration of GNR–PEG–CDs was raised to 50 µg mL^−1^, survival of the cell dropped to only about 30%. Further increasing of the concentration did not result in decrease in viability. To visualize the anticancer effect of the GNR–PEG–CDs material, the dead and live HeLa cells were stained with propidium iodide (PI) and Calcein-AM, respectively. As shown in Figure 6c, there was no cell death in the control group (PBS) and GNR–PEG–CDs without laser irradiation group because almost all cells display green fluorescence, an indication of biocompatibility of GNR–PEG–CDs. However, upon laser irradiation, all of the cells were killed in the GNR–PEG–CDs sample, as indicated by the intense homogeneous red fluorescence. These results agree with the cell viability data and prove the excellent abilities of this hybrid nanomaterials for PTT.

## 4. Conclusions

In summary, we accomplished the preparation of a novel GNR–PEG–CDs multifunctional nanoplatform through a convenient and facile method. The compact structure presented in this paper is more stable owing to a strong covalent bond between the CDs and the GNRs. As a luminescent nanomaterial, it exhibited ideal characteristics for dual-modal bioimaging and photothermal performance in vitro. Upon irradiation with NIR light, GNR–PEG–CDs could efficiently inhibit the growth of HeLa cells, and the distribution of the nanomaterial can be imaged via fluorescence microscopy, as well as a fluorescence lifetime imaging technique. It is worth mentioning that this nanohybrid is highly biocompatible at higher concentrations, and it can produce visible effects even at lower concentrations. Therefore, the hybrid GNR–PEG–CDs demonstrated intriguing potential for biomedical applications. In the future, we are committed to preparing CDs with high QYs and optimizing the distance of GNR and CDs to enhance the emission. Meanwhile, this new biocompatible nanohybrid can be exploited in both multimodal imaging and synergistic therapeutics in vivo, which may provide new opportunities for cancer diagnostics and therapeutics.

## Supplementary Materials

The following are available online at http://www.mdpi.com/2079-4991/8/9/706/s1, Figure S1: (A) The test curve of Zeta potential: (a) GNR, (b) GNR–PEG_400_, (c) GNR–PEG_600_, (d) GNR–PEG_1000_. (B) The fit curve of Zeta potential of (a), (b), (c), (d); Figure S2: UV-vis spectra of GNR and GNR–PEG (molecular weights of PEG are 400, 600 and 1000, respectively); Figure S3: Histograms and Gaussian fittings of particle size distribution of the CDs; Figure S4: UV-vis spectra of GNR, GNR-PEG_1000_ and GNR-PEG-CDs; Figure S5: TEM of the hybrid (100 µg mL^−1^) after irradiation (a) and ultrasonic (b) for 10 min.
