# Supplementary material for: Gold Rod-Polyethylene Glycol-Carbon Dot Nanohybrids as Phototheranostic Probes
Source: Nanomaterials (Basel). 2018 Sep 10;8(9):706. doi: 10.3390/nano8090706 (PMC6165167; doi:10.3390/nano8090706)
Supplement: Supplementary file 1 [file nanomaterials-08-00706-s001.pdf]

nanomaterials

Supporting Information

Article

Gold Rod-Polyethylene Glycol-Carbon Dot Nanohybrids as Phototheranostic Probes

Yuefang Niu [1,3], Guo Ling [1], Li Wang [1,3], Shanyue Guan [1], Zheng Xie [1,*], Eran A. Barnoy [2], Shuyun Zhou [1,*] and Dror Fixler [2,*]

1 Key Laboratory of Photochemical Conversion and Optoelectronic Materials, Technical Institute of Physics and Chemistry, Chinese Academy of Sciences, 29 Zhongguancun East Road, Haidian District, Beijing, 100190, China; niuyuefang15@mails.ucas.ac.cn (Y.N.); lingguo14@mail.ipc.ac.cn (G.L.); wangli165@mails.ucas.ac.cn (L.W.); guanshanyue@mail.ipc.ac.cn (S.G.)
2 Faculty of Engineering and Institute of Nanotechnology and Advanced Materials, Bar-Ilan University, Ramat-Gan 52900, Israel; eabnoy@gmail.com
3 University of Chinese Academy of Sciences, 19A Yuquan Road, Shijingshan District, Beijing, 100049, China
* Correspondence: zhengxie@mail.ipc.ac.cn (Z.X.); zhou_shuyun@mail.ipc.ac.cn (S.Z.); Dror.Fixler@biu.ac.il (D.F.); Tel.: +86-10-82543636 (Z.X.); +86-10-82543551 (S.Z.); +972-3-531-7598 (D.F.)

Quantum yields (QYs) measurements

We chose rhodamine B (QY=89% at 495 nm excitation in ethanol) as the reference sample to measure the QY of the CDs. The QY of the sample was calculated according to the following equation:

$$\varphi = \varphi' \times \frac{A'}{I'} \times \frac{I}{A} \times \frac{n^2}{n'^2}$$

where ϕ is the QY of the testing sample, I is the testing sample's integrated emission intensity, n is the refractive index (1.33 for water and 1.36 for ethanol), and A is the optical density. The superscript "′" refers to the referenced fluorescent dyes of known QYs. To obtain more reliable results, a series of solutions of CDs and referenced fluorescent dyes were prepared with concentrations adjusted so that the optical absorbance values were between 0 – 0.1 at 495 nm. The PL spectra were measured and the PL intensity was integrated. After linear fitting, we obtain the slope for the CDs and the referenced dye, and we can calculated the QY of the CDs according to the abovementioned equation.

Calculation of the photothermal conversion efficiency

The photothermal conversion efficiency, η_T, was calculated using Equation (1) according to Roper's method [1]:

$$\eta_T = \frac{hA(T_{max} - T_{amb}) - Q_0}{I(1 - 10^{-A_\lambda})} \tag{1}$$

where h is the heat transfer coefficient, A is the surface area for radiative heat transfer. T_{max} is the maximum system temperature, T_{amb} is the ambient surrounding temperature and the (T_{max} - T_{amb}) was 32.9 ºC according to Figure 3c. I is the laser power (in units of W, 1.5 W) and A_λ is the absorbance of GNR-PEG-CDs (0.740) at 808 nm. Q_0 was the heat input (in units of W) derived from light absorption by the solvent, which was measured independently to be 0.082 W. Further, hA was determined by measuring the rate of temperature drop after removing the laser, obtained from Figure 3d. The value of hA is based on a dimensionless driving force temperature, θ, and a sample system time constant τ_s, according to Equations (2), (3), and (4).

$$\theta = \frac{T - T_{amb}}{T_{max} - T_{amb}} \tag{2}$$

$$\tau_s = \frac{m_D C_D}{hA} \tag{3}$$

$$t = -\tau_s \ln(\theta) \tag{4}$$

where τ_s was determined to be 420 s for this composite by applying the linear time data from the cooling stage versus the negative natural logarithm of the driving force temperature (Figure 3d). The m_D and C_D are, respectively, the mass (1.5 g) and heat capacity (4.2 J g^{-1}) of deionized water (the solvent). Therefore, the photothermal conversion efficiency can be calculated to be 33.5% for GNR-PEG-CDs.

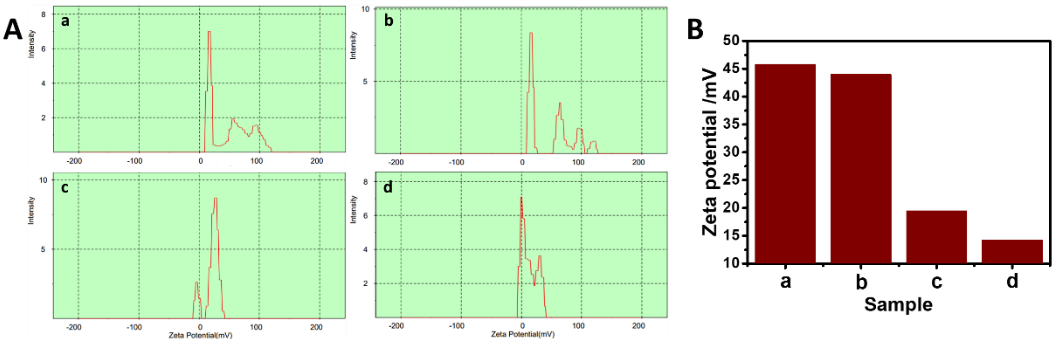

Figure S1. (**A**) The test curve of Zeta potential: (**a**) GNR, (**b**) GNR–PEG$_{400}$, (**c**) GNR–PEG$_{600}$, (**d**) GNR–PEG$_{1000}$. (**B**) The fit curve of Zeta potential of (a), (b), (c), (d).

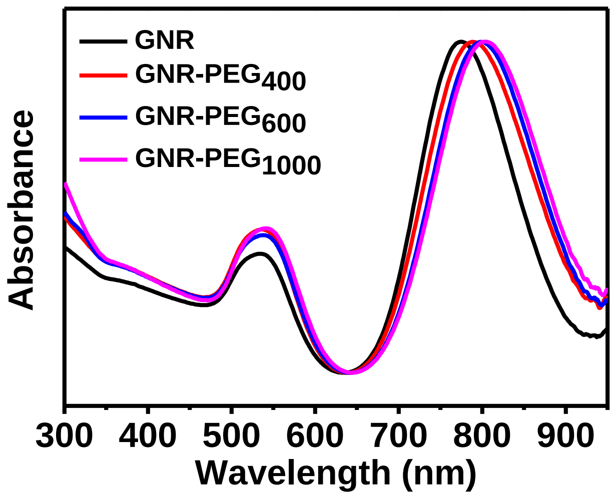

Figure S2. UV-vis spectra of GNR and GNR–PEG (molecular weights of PEG are 400, 600 and 1000, respectively).

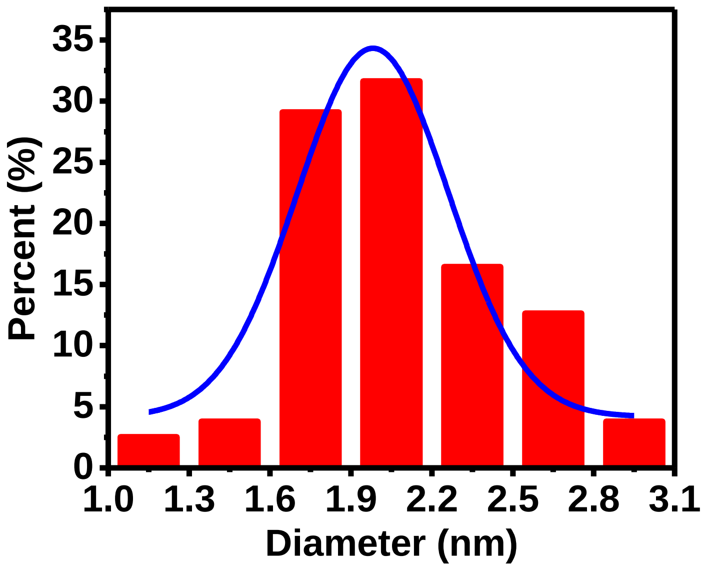

Figure S3. Histograms and Gaussian fittings of particle size distribution of the CDs.

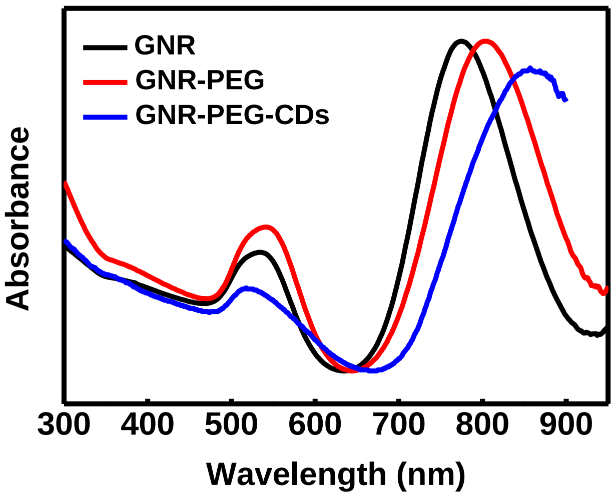

Figure S4. UV-vis spectra of GNR, GNR-PEG1000 and GNR-PEG-CDs.

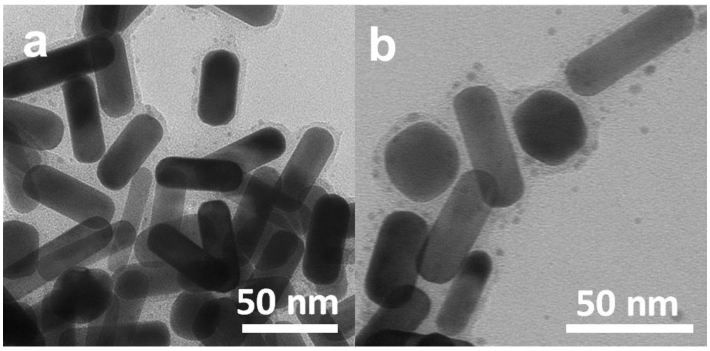

Figure S5. TEM of the hybrid (100 μg mL^{-1}) after irradiation (**a**) and ultrasonic (**b**) for 10 min.

Reference:

1. Roper, D.K.; Ahn, W.; Hoepfner, M. Microscale heat transfer transduced by surface plasmon resonant gold nanoparticles. *J. Phys. Chem. C* **2007**, *111*, 3636–3641.

© 2018 by the authors. Submitted for possible open access publication under the terms and conditions of the Creative Commons Attribution (CC BY) license (http://creativecommons.org/licenses/by/4.0/).
